# Implementation and comparison of kernel-based learning methods to predict metabolic networks

**DOI:** 10.1007/s13721-016-0134-5

**Published:** 2016-07-15

**Authors:** Abiel Roche-Lima

**Affiliations:** Collaboration Center for Research in Health Disparities, Medical Science Campus, University of Puerto Rico., PO Box 365067, San Juan, PR 00936-5067 USA

**Keywords:** Network prediction, Metabolic pathways, Machine learning, Kernel methods

## Abstract

Metabolic pathways can be conceptualized as the biological equivalent of a data pipeline. In living cells, series of chemical reactions are carried out by different proteins called enzymes in a stepwise manner. However, many pathways remain incompletely characterized, and in some of them, not all enzyme components have been identified. Kernel methods are useful in many difficult problem areas, such as document classification and bioinformatics. Specifically, kernel methods have been used recently to predict biological networks, such as protein–protein interaction networks and metabolic networks. In this paper, we implement and compare different methods and types of data to predict metabolic networks. The methods are Penalized Kernel Matrix Regression (PKMR) and pairwise Support Vector Machine (pSVM). We develop several experiments using these methods with sequence, non-sequence, and combined data. We obtain better accuracy when the sequence data are used in both methods. Whereas when the methods are compared using the same type of data, the pSVM approach shows better accuracy. The best results are obtained with pSVM using all combined kernels.

## Introduction

Biochemical pathways are chemical reactions in the cell where enzymes catalyse reactions to produce other compounds based on substrates. For example, in the metabolic pathway that involves glycolysis, the glucose is broken down into smaller products, such as carbon dioxide and water (Luo et al. 2007). Finding the enzymes involved in the reactions and their interactions is still a very challenging topic. The development of pathway databases, such as KEGG (Kanehisa et al. 2008) and EcoCyc (Latendresse et al. 2012), has increased the current knowledge about metabolic networks. Using these databases, methods based on gene annotations are used to predict metabolic networks (Latendresse et al. 2012; Karp et al. 2011). However, current genome annotation pipelines may fail to assign identities correctly to score genes and to detect other genes altogether. Thus, metabolic network prediction algorithms using current genome annotation pipelines may predict inaccurate interactions, for example, the Pathway Tools described by Karp et al. (2011).

To infer metabolic networks, supervised learning approaches have been developed in the framework of kernel methods by Kotera et al. (2013), such as Support Vector Machines (SVMs). While SVMs are a classical paradigm in machine learning, they cannot be directly applied to the biological network inference problems, since the goal is to predict pair of genes (Ben-Hur and Noble 2005). Thus, the pairwise Support Vector Machine (pSVM) approach is used instead (Oyama and Manning 2004). Vert et al. (2007) and Kashima et al. (2010) apply pSVM methods to predict metabolic networks, but only combine non-sequence data. In addition, Roche-Lima et al. (2014) use sequence kernels (i.e., PRK—Pairwise Rational Kernels) combined with SVM methods and obtain good accuracy values and execution times, but do not compare with non-sequence kernels.

There are other supervised learning algorithms, such as Kernel Canonical Correlation Analysis (KCCA) (Yamanishi et al. 2004) and Penalized Kernel Matrix Regression (PKMR) (Yamanishi and Vert 2007), which are computationally more efficient, but they lack the ability to give precise predictions. In addition, these algorithms have only been reported in the literature using non-sequence kernels (Yamanishi 2010; Kotera et al. 2012).

In our research, we consider these problems, implementing methods to predict metabolic networks based on raw data directly related to the sequence information (e.g., nucleotides and protein sequences). We hypothesize that sequence kernels, created from raw sequence data, will be more precise that non-sequence kernels to predict metabolic networks. We then implement two of the supervised learning methods (i.e., PKMR and pSVM), and for first time, we compare these two methods combined with sequence and non-sequence kernels.

## Materials and methods

### Metabolic networks

Metabolic networks were represented as a graph, where vertices (nodes) were the enzymes, and the edges (branches) were the enzyme–enzyme relations (proteins catalyzing two continuous reactions in a pathway). Traditionally, metabolic pathway representations considered enzymes as vertices, and metabolites as edges. To avoid confusion, our graphs represented interactions between pairs of enzymes as discrete data points similar to Yamanishi (2010). An example of the graph representation can be seen in (Roche-Lima et al. 2014, Fig. 2).

### Data

We used information of the yeast *Saccharomyces cerevisiae* (Sikorski and Hieter 1989) taken from the KEGG pathway databases (Kanehisa et al. 2008). This species was selected, because it was a well-studied organism with several defined models to predict biological networks. Moreover, other kernel methods had been described and tested using data from this species (Ben-Hur and Noble 2005; Kashima et al. 2010; Yamanishi 2010). As a training set, we used 5149 interactions from 755 known genes. A graph was built based on these interactions (training set) as a representation of the metabolic networks of the yeast *Saccharomyces cerevisiae*. Then, this graph and other related data sets were converted into kernels.

Kernels allowed working in a unified mathematical framework across different types of data. A kernel was a measure of similarity that satisfied the additional condition of being a dot product in some feature space (see Scholkopf and Smola 2002 for details). Data were represented as a positive definite kernel *K* that was a symmetric function $K :\;X^{ 2} \to {\mathbb{R}}$ that satisfied $\sum\nolimits_{i,j}^{n} {a_{i} a_{j} K\left( {Xx_{i} X_{j} } \right)}$ and $(a_{ 1} ,\;a_{ 2} , \ldots ,\;\;a_{n} ) \in {\mathbb{R}}^{n}$, where *X* was the set of entities.

#### Non-sequence data

In our context, non-sequence kernels manipulate data that were binary or numerical. We used three different types of non-sequence data, i.e., gene expression, gene localization, and phylogenetic data. All these data have been used in other research as kernels (Vert et al. 2007; Kashima et al. 2010; Yamanishi 2010). Gene expression data were obtained by Yamanishi (2010) using the results from 157 microarray experiments (Spellman et al. 1998; Eisen et al. 1998). Each gene was associated with a 157-element numerical vector that represented the results from the experiments. Gaussian Radial Bases Function (RBF) Kernel was used to manipulate this data, and we defined the same parameters that Yamanishi (2010) used in their experiments. We denoted the final kernel as *k*_exp_.

The gene localization data were represented as a 23-element binary vector for each gene, following Yamanishi (2010). A total of 23 intracellular localizations were defined (e.g., mitochondrion, Golgi, nucleus, and others). The value was 1, if the gene was present in the intracellular localization or 0 otherwise. Similar to Yamanishi (2010), we used the linear kernel applied to these data with the same parameters. We denoted this kernel as *k*_loc_.

The phylogenetic profile data were obtained from 145 organisms, which describe the set of orthologous genes. These organisms were selected based on the criteria defined in Yamanishi (2010). Each gene was associated with a 145-element binary vector. The value was 1, if the gene was present in this organism or 0 otherwise. A Gaussian RBF kernel was used to compute this data with the same parameters used by Yamanishi (2010). This final kernel was denoted as *k*_phy_.

#### Sequence data

Sequence kernels defined similarities over finite sequences of symbols with different lengths. The sequence data were then converted to sequence kernels. In our research, we used three sequence kernels, Pfam, Motif, and Spectrum, defined by Ben-Hur and Noble (2005). We chose these sequence kernels to be able to compare our results with the previous published works, such as Yu et al. (2010); Ben-Hur and Noble (2005); Roche-Lima et al. (2014), and Allauzen et al. (2008).

The Pfam kernel (Gomez et al. 2003) was computed based on a set of Hidden Markov Models (HMMs), where each gene that codes for an enzyme was compared with every HMM in the Pfam database. The *E* value statistics were obtained as features for the 13,672 domain HMMs in the Pfam version 26.0 (Punta et al. 2012). Thus, each protein was represented by a vector of 13,672 log *E* values, and the kernel was computed based on these vectors (see Allauzen et al. (2008) for more details). We denoted this kernel as *k*_pfam_.

The Motif kernel (Ben-Hur and Brutlag 2003) was also used. It was obtained by calculating how many times a discrete sequence motif matched each of the protein sequences. The eMotif database (Huang and Brutlag 2001) was used to extract the discrete sequence motifs. A vector of *E* values was associated for each of the proteins (genes coding for the proteins). The kernel was finally computed as dot products of those vectors (see Ben-Hur and Noble 2005 for more details). The kernel was called *k*_motif_.

Finally, the Spectrum kernel defined by Leslie et al. (2004) was also considered. This kernel represented sequence similarities by counting how many times an *n*-gram (*k*_mer_) appeared in each of the pairs of sequences. Each gene had an associated featured vector of *n*-gram counts (we considered *n* = *3*). Similar to the data above, the kernel was computed to represent the dot products using the associated feature vectors. We denoted this kernel as *k*_ngram_.

#### Combined data

We also computed the linear combination of the kernels described above, representing the heterogeneous data combination. We used different types of data to predict metabolic networks. $K_{1} , \ldots ,K_{n}$ were the kernels that represented the data, so $K_{n}$ corresponded to the *n*-th data set. Yamanishi (2010) mentioned the advantages of considering the linear combination as weighted sum of kernels, i.e., $\sum\nolimits_{n = 1}^{N} {W_{n} K_{n} }$, where $W_{n}$ was a weight (real coefficient) associated to the kernel $K_{n}$. The coefficients should be related to the importance of the data set *n* for the prediction method. In our research, we considered the weights ($W_{n}$) as the accuracy values obtained during the inference process using the individual kernel $K_{n}$, i.e., ROC score Yamanishi et al. (2005). In future studies, weight values may be computed in different ways.

### Methods

We used kernel-based supervised learning network inference methods to predict biological networks based on kernel frameworks. First, part of the network (with known interactions—training set) was used during the learning inference process to obtain the model. Second, new interactions were predicted using the model. In machine learning, supervised classifications are a classical paradigm. However, it could not be applied directly to the problem of network inference, because our goal was to predict relations between pairs of nodes, not individual nodes (Yamanishi 2010). Therefore, we first define the pairwise kernel and, later, the methods PKMR and pSVM.

#### Pairwise kernels

The kernels described in the sections above provide similarities between simple enzymes. In our experiments, we used a different type of kernel called pairwise kernel (Pahikkala et al. 2012; Brunner et al. 2012) that provide similarity measures for pairs of entities. The general pairwise kernel was represented as $K:\;(X \times \;X) \, (X\; \times \;X) \to \varvec{R}$, where $X$ is a set of vertices (enzymes) and $\varvec{R}$ is a set of real values. In this research, we used the Pairwise Tensor Product Kernel or Kronecker Kernel (Basilico and Hofmann 2004; Oyama and Manning 2004; Ben-Hur and Noble 2005) that is computed as $K\left( {\left( {X_{1} ,X_{2} } \right), \left( {X^{\prime}_{1} ,X^{\prime}_{2} } \right)} \right) = k'\left( {X_{1} ,X^{\prime}_{1} } \right)k'\left( {X_{2} ,X^{\prime}_{2} } \right) + k'\left( {X_{1} ,X^{\prime}_{2} } \right)k'\left( {X_{2} ,X^{\prime}_{1} } \right)$, where $k'$ is a simple kernel and $X_{1} ,\;X_{2} ,\;X'_{1} ,\;X'_{2}$ are the enzymes ($k'$ represent any of the kernel described in the previous sections).

#### Penalized kernel matrix regression (PKMR)

Kernel Matrix Regression methods were based on the supervised graph inference framework to predict metabolic networks with metric learning. A formalism of the problem can be defined as follows:

given an undirected graph $\varGamma = (V, \, E)$, with a set of vertices $V = (V_{1} ,\; V_{2} , \ldots , \;V_{n} )$ and a set of edges $E \subset \left( {V\; \times \; V} \right),$

then, for an additional set of vertices $V^{\prime}\; = \;\;V_{1}^{\prime} , \;V_{2}^{\prime} , \ldots ,\; V_{n}^{\prime} ,$ the goal was to infer the set of new edges $E^{\prime} \subset \;\;V^{\prime}\; \times \;\left( {V\; + \; V^{\prime}} \right)\; \cup \;\left( {V\; + \;V^{\prime}} \right)\; \times \;V',$ that involved the additional vertices in $V^{\prime}$.

Yamanishi et al. (2005) described methods to solve this problem, such as KCCA, PKMR, Kernel Matrix Completion, and Expectation-Maximization algorithms. He obtained as a result that the method with the best accuracy was PKMR (Yamanishi and Vert 2007), a modified version of Kernel Matrix Regression method.

In our research, we implemented PKMR using the R library (R Core Team 2013). To make the data compatible with the pSVM method, the chemical compatibility network was not taken into consideration (Yamanishi 2010), since we aimed to compare data directly related to the genes. Future implementations may include this information.

#### Pairwise support vector machine (pSVM)

pSVM methods classified whether a pair ($x_{1} ,\;y_{1}$) belonged to the same category or to a different one. Then, while SVM methods classified simple entities, pSVM methods classified pairs of entities. pSVM was defined by Brunner et al. (2012) as follows:

given a training data set $((x_{i} ,\;\;y_{i} ),\;d_{i} ),\;d_{i}$ with binary classification values (i.e., $(x_{i} ,\;y_{i} )$ classified as +1 or $(x_{i} ,\;y_{i} )$ classified as −1), *i* = 1, …, *n* and the function $\varPhi ,$

then, a pSVM method found an optimal hyperplane, i.e., $w^{T} \varPhi \left( {x_{i} ,y_{i} } \right) + b = 0,$ where the points were separated into two categories.

We implemented programs to apply pSVM to predict the metabolic networks with our data sets, using LIBSVM (Chang and Lin 2011) and Python Machine Learning (PyML) (Ben-Hur et al. 2008) libraries.

### Experiments

We developed six groups of experiments using different data and methods (see Table 1 for more details).

**Table 1:** Experiments are grouped by methods (experiment I, II, III—PKMR and experiment IV, V, VI—pSVM) and by type of data (I, IV—non-sequence data, II, V—sequence data, and III, VI—combined data)

Experiment	Methods	Type of kernel
I	PKMR	Non-sequence (described in Sect. 2.2.1)
II	PKMR	Sequence (described in Sect. 2.2.2)
III	PKMR	Combined sequence and non-sequence (described in Sect. 2.2.3)
IV	pSVM	Non-sequence (described in Sect. 2.2.1)
V	pSVM	Sequence (described in Sect. 2.2.2)
VI	pSVM	Combined sequence and non-sequence (described in Sect. 2.2.3)

For evaluation, we used the area under the ROC curve (AUC score) (Gribskov and Robinson 1996) to measure the accuracy. It was defined as a function of the rates of true-positives (predicted enzymes pairs were present in the data set) and false-positives (predicted protein pairs were absent in the data set). A stratified cross-validation procedure was used with fold equal to 10 (tenfold cross-validation) (Kohavi et al. 1995). We also collected execution times (Time s). All experiments were run using a computer with a microprocessor Intel i7CORE and RAM memory of 8 MB.

In addition, we computed the 95 % confidence intervals (CIs) for average AUC scores. We used a distribution-independent technique proposed by Cortes and Mohri (2005). As they described, the variance depends on the number of positive and negative examples in the training set and the number of errors during the classification process. In our case, the training set consisted in 2575 positive and 2574 negative interactions, out of 5149 total interactions. The errors in the classification process ranged between 750 and 1851.

## Results and discussion

### Comparing data

When we compare sequence and non-sequence data, within the same supervised learning method, better accuracy values are obtained with the sequence kernel (see Table 2, Experiments II–PKMR–Sequence and V–pSVM–Sequence). For example, in the PKMR method, the accuracy value is improved from AUC = 0.503 (the lowest value in experiment I–PKMR–Non-Sequence kernel) to AUC = 0.821 (the highest value in experiment II–PKMR–Sequence kernel). This proves our hypothesis about better accuracy values when sequence kernels are used, since errors from the genome annotation process are bypassed. However, the execution times for the methods using sequence kernels are more than doubled when they are compared with non-sequence kernels (i.e., Time = 240 s—the lowest time in experiment I–PKMR–Non-Sequence versus Time = 530 s—the highest time in experiment II–PKMR–Sequence). This is because computing sequence kernels (i.e., *k*_pfam_ *, k*_motif_, and *k*_mer_) consume more computational resources.

**Table 2:** Results collected during the experiments

Experiment	Predictor kernel	AUC score	Time s	Confidence intervals
I–PKMR–Non-Sequence	*k*_exp_	0.660	300	[0.655, 0.665]
	*k*_loc_	0.503	240	[0.499, 0.507]
	*k*_phy_	0.775	240	[0.771, 0.779]
	*k*_exp_ + *k*_loc_ + *k*_phy_	0.755	350	[0.752, 0.759]
	*w*_1_ *k*_exp_ + *w*_2_ *k*_loc_ + *w*_3_ *k*_phy_	0.799	420	[0.791, 0.807]
II–PKMR–Sequence	*k*_pfam_	0.797	450	[0.793, 0.801]
	*k*_motif_	0.782	430	[0.778, 0.786]
	*k*_mer_	0.725	420	[0.720, 0.731]
	*k*_pfam_ + *k*_motif_ + *k*_mer_	0.817	480	[0.811, 0.823]
	*w*_4_ *k*_pfam_ + *w*_5_ *k*_motif_ + *w*_6_ *k*_mer_	0.821	530	[0.818, 0.824]
III–PKMR–Combined (sequence and non-sequence)	*k*_phy_ + *k*_pfam_	0.812	470	[0.809, 0.816]
	*k*_exp_ + *k*_loc_ + *k*_phy_ + *k*_pfam_ + *k*_motif_ + *k*_mer_	0.831	610	[0.828, 0.834]
	*w*_1_ *k*_exp_ + *w*_2_ *k*_loc_ + *w*_3_ *k*_phy_ + *w*_4_ *k*_pfam_ + *w*_5_ *k*_motif_ + *w*_6_ *k*_mer_	0.840	720	[0.831, 0.849]
IV–pSVM–Non-Sequence	*k*_exp_	0.791	9020	[0.786, 0.796]
	*k*_loc_	0.696	7800	[0.692, 0.700]
	*k*_phy_	0.802	7980	[0.797, 0.807]
	*k*_exp_ + *k*_loc_ + *k*_phy_	0.818	10,100	[0.812, 0.824]
	*w*_1_ *k*_exp_ + *w*_2_ *k*_loc_ + *w*_3_ *k*_phy_	0.877	10,121	[0.871, 0.883]
V–pSVM–Sequence	*k*_pfam_	0.887	12,060	[0.879, 0.895]
	*k*_motif_	0.868	12,000	[0.859, 0.877]
	*k*_mer_	0.840	11,760	[0.836, 0.844]
	*k*_pfam_ + *k*_motif_ + *k*_mer_	0.898	12,220	[0.891, 0.905]
	*w*_4_ *k*_pfam_ + *w*_5_ *k*_motif_ + *w*_6_ *k*_mer_	0.910	12,800	[0.901, 0.919]
VI–pSVM–Combined (Sequence and non-sequence)	*k*_phy_ + *k*_pfam_	0.890	12,100	[0.882, 0.898]
	*k*_exp_ + *k*_loc_ + *k*_phy_ + *k*_pfam_ + *k*_motif_ + *k*_mer_	0.939	13,420	[0.935, 0.944]
	*w*_1_ *k*_exp_ + *w*_2_ *k*_loc_ + *w*_3_ *k*_phy_ + w_4_ *k*_pfam_ + w_5_ *k*_motif_ + w_6_ *k*_mer_	0.940	14,010	[0.934, 0.946]

These are AUC score (area under the ROC curve as accuracy), time s (Execution times in seconds), and confidence intervals

The best results are obtained with the kernels that represent the combined heterogeneous data within the same supervised learning method, i.e., Table 2, experiment III–PKMR–Combined and VI–pSVM–Combined. In this case, for the PKMR method, the accuracy is improved from AUC = 0.797 (i.e., the best accuracy using the simple kernel—*k*_pfam_ in Experiment II) to AUC = 0.840 (i.e., weighted kernel: $w_{1} k_{ \exp } + w_{2} k_{\text{loc}} + w_{3} k_{\text{phy}} + w_{4} k_{\text{pfam}} + w_{5} k_{\text{motif}} + w_{3} k_{\text{mer}}$ in Experiment III). Likewise, when we use only the Pfam*—k*_*pfam*_ kernel (AUC = 0.797 in experiment II), the best accuracy is obtained. Then, we also test this simple sequence kernel combined with other simple kernels. The best results are obtained combining the Pfam*—k*_pfam_ and phylogenetic—*k*_phy_ kernels (see the accuracy values of *k*_phy_ + *k*_pfam_ kernel in Table 2, experiment III–PKMR–Combined and VI-pSVM–Combined). This result coincides with Allauzen et al. (2008), where they stated “the importance of the phyletic retention feature as a possible reason for the superior performance of the combined kernel compared with Pfam alone”.

### Comparing methods

As can be seen in Table 2, pSVM methods (experiment IV–pSVM–Non-Sequence, V–pSVM–Sequence, and VI–pSVM–Combined) outperform the precision values of PKMR method (experiment I–PKMR–Non-Sequence, II–PKMR–Sequence and III–PKMR–Combined). For example, using the PKMR method, the AUC score for *k*_exp_ (Experiment I–PKMR–Non-Sequence) is 0.660 compared to 0.791 (experiment III–PKMR–Combined). However, the execution times are considerably increased for pSVMs (see Table 2 Times values for experiments I–PKMR–Non-Sequence and II–PKMR–Sequence in comparison with experiments IV–pSVM–Non-Sequence and V–pSVM–Sequence). Processing pSVM involves more computational resources than PKMR methods; however, better accuracy values are obtained. In all the cases, the confidence intervals are above the behaviour of a random classifier.

**Fig. 1:**
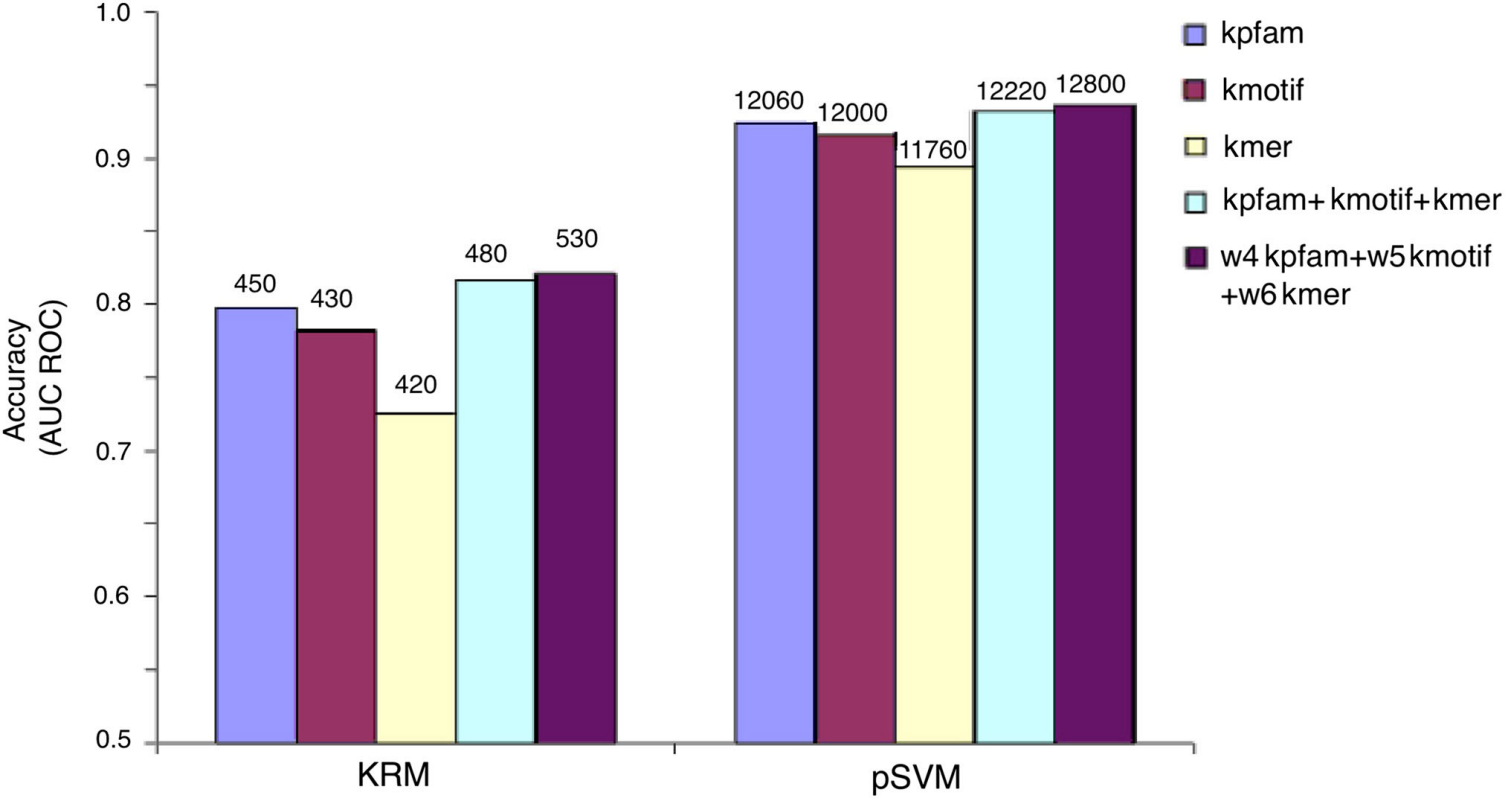
Comparison of the methods (PKMR—Penalized Kernel Matrix Regression and pSVM—pairwise Support Vector Machine) for the sequence data kernels, related to accuracy and execution times

Figure 1 represents the results for both methods (PKMR and pSVM) using only sequence kernels. Although the most time consuming method is pSVM, it provides an important improvement in the accuracy values [the peaks are reached combining the sequence kernel (*k*_pfam_ + *k*_motif_ + *k*_ngram_) and pSVM method]. Yamanishi (2010) mentions these expected high processing times for SVM methods, but never tested them to evaluate how the accuracy could be improved. Roche-Lima et al. (2014) use a different representation of the sequence kernel and decrease execution time of the sequence kernel computation; however, they still use existing SVM methods. In addition, the accuracy values obtained in this research are better than the values reported by Roche-Lima et al. (2014). Thus, we consider that pSVM implementation can be optimized to obtain better processing time and to maintain these good accuracy values. Likewise, sequence kernel representations can be also optimized to combine with pSVM methods to improve both performance and accuracy.

## Conclusion

We developed, for the first time, experiments using sequence data with PKMR and pSVM methods to predict metabolic networks. We proved that the best accuracy values were obtained using sequence kernels. This was because other tools to predict metabolic networks were based on the gene annotations (Latendresse et al. 2012; Karp et al. 2011). As we used raw sequence data (represented as sequence kernels), it bypassed the annotations and the errors associated with these steps.

We also proved that pSVM methods were more precise than PKMR methods. The best accuracy values were obtained when pSVM methods were combined with sequence kernels. However, pSVM methods were very expensive in terms of computational resources, such as execution times. pSVM methods required even more computational resources when using sequence kernels.

In future works, pSVM method can be optimized using other implementations, such as Dual Coordinate Descent algorithm combined with rational kernels to manipulate sequence data (Allauzen et al. 2011). As well, a parallel implementation could be used to improve performance (Tyree et al. 2014).
